# Comparison of structural characteristics and molecular markers of rabbit skin, pig skin, and reconstructed human epidermis for an ex vivo human skin model

**DOI:** 10.1007/s43188-023-00185-1

**Published:** 2023-05-04

**Authors:** Chanyang Uhm, Haengdueng Jeong, Su Hyon Lee, Jae Sung Hwang, Kyung-Min Lim, Ki Taek Nam

**Affiliations:** 1 Severance Biomedical Science Institute, Brain Korea 21 PLUS Project for Medical Science, Yonsei University College of Medicine, 50-1 Yonsei-Ro, Seodaemun-Gu, Seoul, 03722 Republic of Korea; 2 GLP Center, Biosolution Co., Ltd., Seoul, 06746 Republic of Korea; 3 Genetics and Biotechnology, College of Life Science, Kyung Hee University, Yongin, 17104 Republic of Korea; 4 College of Pharmacy, Ewha Womans University, 52 Ewhayeodae-Gil, Seodaemun-Gu, Seoul, 03760 Republic of Korea

**Keywords:** Skin irritation testing, Ex vivo skin, Epidermis, Reconstructed human epidermis, Porcine

## Abstract

The Organization for Economic Co-operation and Development approved a reconstructed human epidermis (RHE) model for *in vitro* skin irritation and corrosion tests as an alternative to animal testing for cosmetics, which has been banned in the European Union since 2013. However, RHE models have several limitations, such as high manufacturing costs, a loose skin barrier, and inability to simulate all cellular and non-cellular components of the human epidermis. Therefore, new alternative skin models are needed. Ex vivo skin models have been suggested as promising tools. Here, we investigated the structural similarities in the epidermis of pig and rabbit skin, a commercial RHE model (Keraskin), and human skin. To compare the structural similarity, the thickness of each epidermal layer was compared using molecular markers. Among the candidate human skin surrogates, the epidermal thickness of the pig skin was the most similar to that of human skin, followed by rabbit skin and Keraskin. Keraskin showed thicker cornified and granular layers than human skin, while rabbit skin displayed thinner layers. Moreover, the proliferation indices of Keraskin and rabbit skin were higher than those of human skin, whereas the proliferation index of the pig skin was similar to that of human skin. Some or none of the human skin barrier proteins FLG, CLDN1, and CDH1 were expressed in pig and rabbit skin, whereas all human proteins were expressed in Keraskin. Collectively, we propose ex vivo pig skin as the most suitable model for skin irritation testing because of its similarity to human skin.

**Supplementary Information:**

The online version contains supplementary material available at 10.1007/s43188-023-00185-1.

## Introduction

With the increasing interest in beauty and health in modern society, cosmetics have become a major industry. Before the approval of new cosmetics products, potential skin hazards should be assessed following a defined chemical exposure. For this purpose, rabbits have been widely used as animal models in the skin irritation test (SIT) based on the Draize eye toxicity test developed in 1944 [1] because of their advantages, such as ease of experimentation and low testing costs. However, in contrast to human skin, rabbit skin is loosely connected to the subcutaneous connective tissue [2], which may explain why rabbits exhibit more sensitive responses to skin irritants than humans, often resulting in an overestimation of a chemical’s potential for skin irritation [3]. Thus, there is little scientific evidence to explain why rabbits continue to be used for the SIT. Furthermore, since 2013, animal experiments with cosmetics have been prohibited in the European Union owing to ethical issues [4], requiring alternative test methods to the rabbit SIT.

Reconstructed human skin (RHS) models have been developed as alternatives to animal models, including reconstructed human epidermis (RHE), full-thickness human skin, and skin organ culture models. The advantage of an RHS model is that it can mimic the function of real human skin *in vivo* because it has a fully differentiated epidermis. The Organization for Economic Co-operation and Development (OECD) has endorsed RHE models for *in vitro* SITs as an alternative to animal experiments [5–7]. However, RHE models also have limitations in that they are expensive, and it is difficult to completely reproduce all components of real human skin.

The pig skin closely resembles the human skin from both histological and physiological aspects [8–10]. In particular, the thickness of the epidermis, cornified layer, and dermal-epidermal thickness ratio were confirmed to be very similar between the human and pig skin [11]. Pig skin also has rete ridges and a pars papillaris, similar to human skin [12]. Importantly, ex vivo pig skin is readily available from slaughterhouses, which can solve animal ethics issues while simultaneously helping to reduce experimental costs [13].

Here, we investigated the structural similarity of the epidermis as another line of evidence for the advantage of ex vivo pig skin as a surrogate model for human skin. We further compared human skin with two other candidate surrogate skin models: Keraskin, a commercial RHE model endorsed in OECD Test Guideline (TG) 439, and rabbit skin. To confirm structural similarity, the thickness of each epidermal layer was compared using molecular markers.

## Methods

### Skin samples

Human skin samples were obtained from healthy donors at Severance Hospital, Yonsei University. All human samples were collected with informed consent for research use, and the study design was approved by the Institutional Review Board (4-2019-0582). Keraskin [14] is a commercially available RHE model, which was obtained from Biosolution Co. (Seoul, Korea). Ex vivo pig skin obtained from Apures Co. (Gyeonggi, Korea) was prepared as previously described [15]. Rabbit skin was obtained from Biosolution Co., Ltd. (Seoul, Korea). The human, rabbit and pig skin were obtained from the chest, back and ear, respectively.

### Immunohistochemistry (IHC)

The epidermis of the skin can be sub-stratified into the stratum basale, stratum spinosum, stratum granulae, stratum lucideum (only in the foot pad), and stratum corneum [16]. The thickness of the stratum, and the granular, spinous, and basal layers constituting the epidermis was measured by IHC staining with specific marker proteins [14], including loricrin (LOR) for the granular layer, keratin 10 (KRT10) for the spinous layer, and keratin 5 (KRT5) for the basal layer. In addition, filaggrin (FLG), claudin (CLDN1), and E-cadherin (CDH1) expressions were compared among the models, which are considered important proteins for the manifestation of skin barrier function in the epidermis. For IHC, the skin samples were sequentially rehydrated with a graded series of ethanol. Next, pH 6.0 antigen retrieval (DAKO, S1699, Santa Clara, CA, USA) was conducted using a high-pressure cooker for 15 min, followed by a cooling phase over 1 h until the solution was fully transparent. After two washes in phosphate-buffered saline (PBS), the sections were incubated in 3% H_2_O_2_ for 30 min to block endogenous peroxidase. After another three washes in PBS, the sections were incubated with a protein block (DAKO, X0909, Santa Clara, CA, USA) for 1–2 h at room temperature in a humidity-controlled chamber. The sections were then incubated with the following primary antibodies overnight at 4 °C: anti-LOR (Abcam, ab24722, 1:2000), anti-KRT10 (Abcam, ab76318, 1:3000), anti-KRT5 (Abcam, ab52635, 1:100,000), anti-MKI67 (Abcam, ab16667, 1:1000), anti-FLG (Novus, NBP1-87,528, 1:1000), anti-CLDN1 (Cell Signaling Technology, 13,255, 1:200), and anti-CDH1 (Cell Signaling Technology, 3195, 1:400). After three washes in PBS, the sections were incubated in horseradish peroxidase (HRP)-labeled anti-rabbit antibody (DAKO, K4003, Santa Clara, CA, USA) for 15 min at room temperature. For development of the HRP-labeled antibody on the sections, DAB (DAKO, K3468, Santa Clara, CA, USA) was diluted and placed on each section for the same period of time. Mayer’s hematoxylin (DAKO, S3309; Santa Clara, CA, USA) was used for counterstaining.

### Histological analysis

All slides were scanned using a virtual microscope scanner (MoticEasyScan One, Hong Kong) at 40 × optical magnification. Thickness was measured in randomly selected fields of view for each model using QuPath software. Epidermal thickness was measured from the beginning of the epidermis to the end of the cornified layer on hematoxylin and eosin (H&E)-stained slides. The thickness of the epidermal layers (cornified, granular, spinous, and basal layers) was measured from the bottom to top direction consistently. The proliferation index was measured in fields divided into four zones for each model using QuPath software. The DAB-positive nucleus percentage was normalized to the total nucleus (hematoxylin + DAB) counts.

### Deposition percentage of the epidermal layer

The deposition percentage of the epidermal layer was calculated as the sum of the average lengths of the granular, spinous, and basal layers of each skin model, and the proportion occupied by each epidermal layer to the sum was calculated.

### Statistical analysis

Statistical analyses were performed using GraphPad Prism v7.0. Statistical significance was determined using unpaired two-tailed Student’s t tests. Data are presented as mean ± standard error of the mean; *p* < 0.05 was considered statistically significant.

## Results

### Morphological comparisons of the epidermis of human, rabbit, and pig skins and the RHE model

**Fig. 1:**
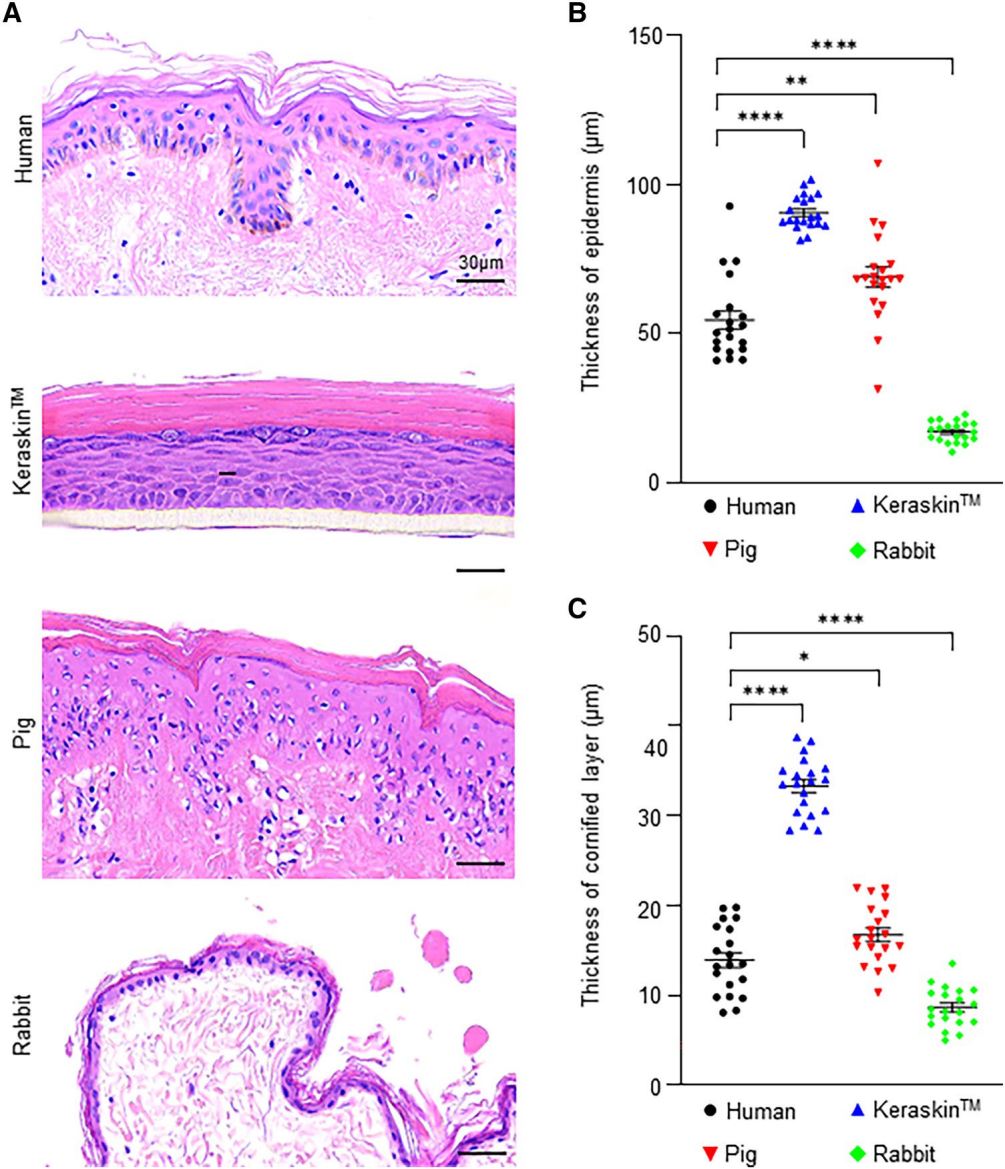
Morphology of the skin epidermis in each model. **A** Hematoxylin and eosin-stained images of human skin, Keraskin, pig skin, and rabbit skin; scale bars = 30 µm. **B** Quantification of the epidermal thickness. Data are presented as the mean ± SEM. **p < 0.01, ****p < 0.0001; unpaired Student’s t test, n = 20. **C** Quantification of the thickness of the cornified layer. Data are presented as the mean ± SEM. *p < 0.05, ****p < 0.0001; unpaired Student’s t test, n = 20

H&E staining was performed to compare the epidermal morphology and epidermal thickness among the models. Human and pig skin showed variable epidermal thickness and rete pegs with a mean thickness of 51.64 µm and 75.47 µm, respectively (Fig. 1A). In contrast to human and pig skin, Keraskin exhibited a flat epidermis with a consistent thickness of 90.33 µm (Fig. 1B). The rabbit skin showed a remarkably thin epidermis, which was approximately one-third of the human skin (Fig. 1A, B).

In the stratum corneum, Keraskin had a stratified cornified layer approximately two-fold thicker than that of the human skin (Fig. 1C). The pig skin exhibited a stratum corneum with a thickness similar to that of human skin. The rabbit skin showed the thinnest stratum corneum of the four models, consistent with the thin epidermis. Overall, the epidermal morphology of the pig skin was the most similar to that of the human skin among the three skin surrogates examined.

### Epidermal sub-layers of human, rabbit, and pig skins and the RHE model

**Fig. 2:**
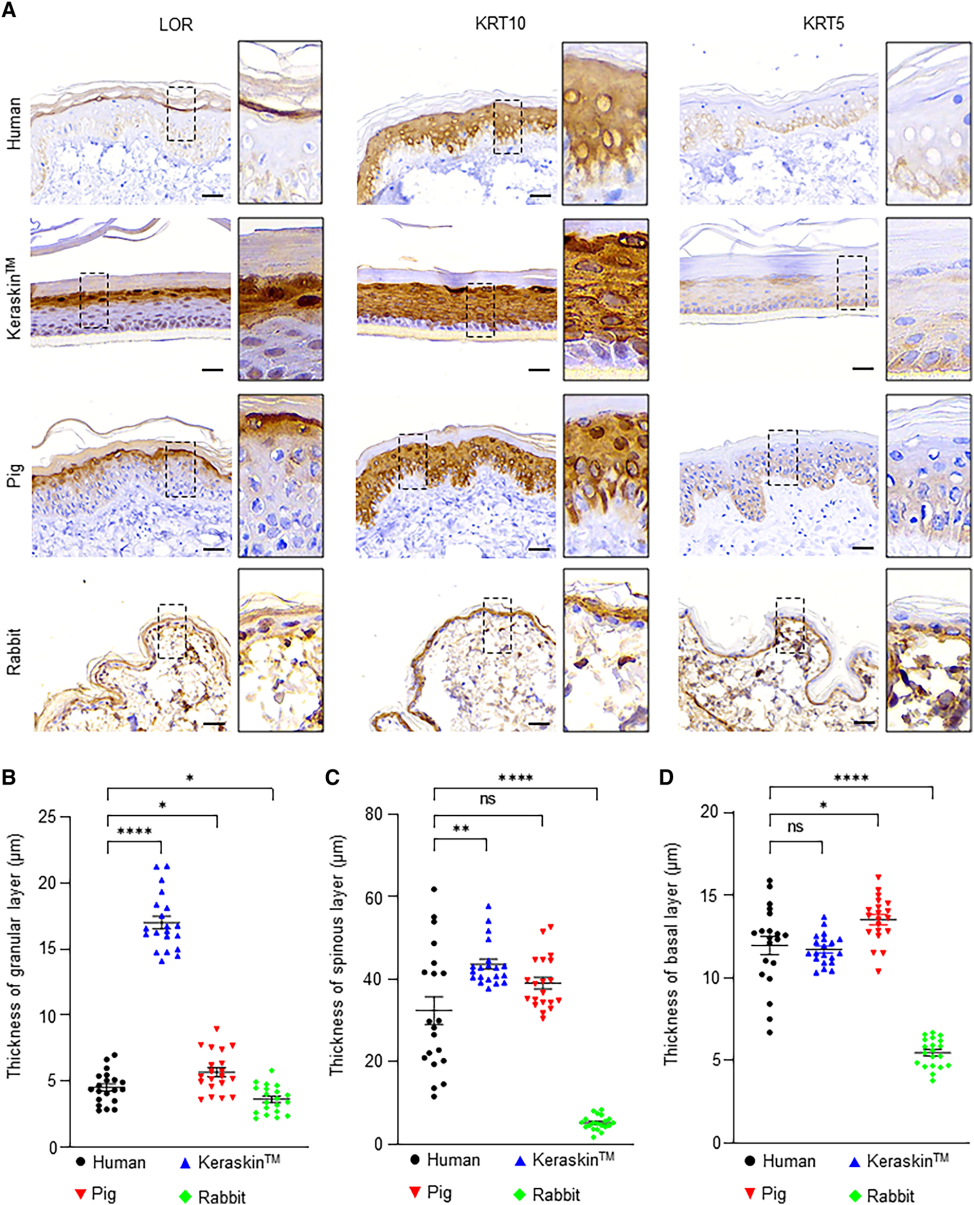
Thickness measurement of each layer of the epidermis in each model. **A** Immunohistochemistry staining images of human skin, Keraskin, pig skin, and rabbit skin for LOR, KRT10, and KRT5. Scale bars = 30 µm. **B** Quantification of the thickness of the granular layer stained by LOR. Data are presented as the mean ± SEM. *p < 0.05, ****p < 0.0001; unpaired Student’s t test, n = 20. **C** Quantification of the thickness of the spinous layer stained by KRT10. Data are presented as the mean ± SEM. ns, not significant (*p* > 0.05); **p < 0.05, ****p < 0.0001; unpaired Student’s t test, n = 20. **D** Quantification of the thickness of the basal layer stained by KRT5. Data are presented as the mean ± SEM. ns, not significant (*p* > 0.05); *p < 0.05, ****p < 0.0001; unpaired Student’s t test, n = 20

IHC staining (Fig. 2A) for LOR showed that Keraskin had the thickest granular layer among the four samples, which was more than four times that of the human skin (Fig. 2B). There were no significant differences in the spinous layer of human and pig skin according to KRT10 expression (Fig. 2C). However, the spinous layer of the rabbit skin was remarkably thin, at approximately one-fifth the thickness of the human skin (Fig. 2C). No significant differences were found in the basal layer of the human skin, pig skin, and Keraskin according to KRT5 staining (Fig. 2D). However, the basal layer of the rabbit skin was the thinnest, corresponding to approximately half that of the human skin (Fig. 2D).

### Thickness of epidermal layers in human, rabbit, and pig skins and the RHE model

**Fig. 3:**
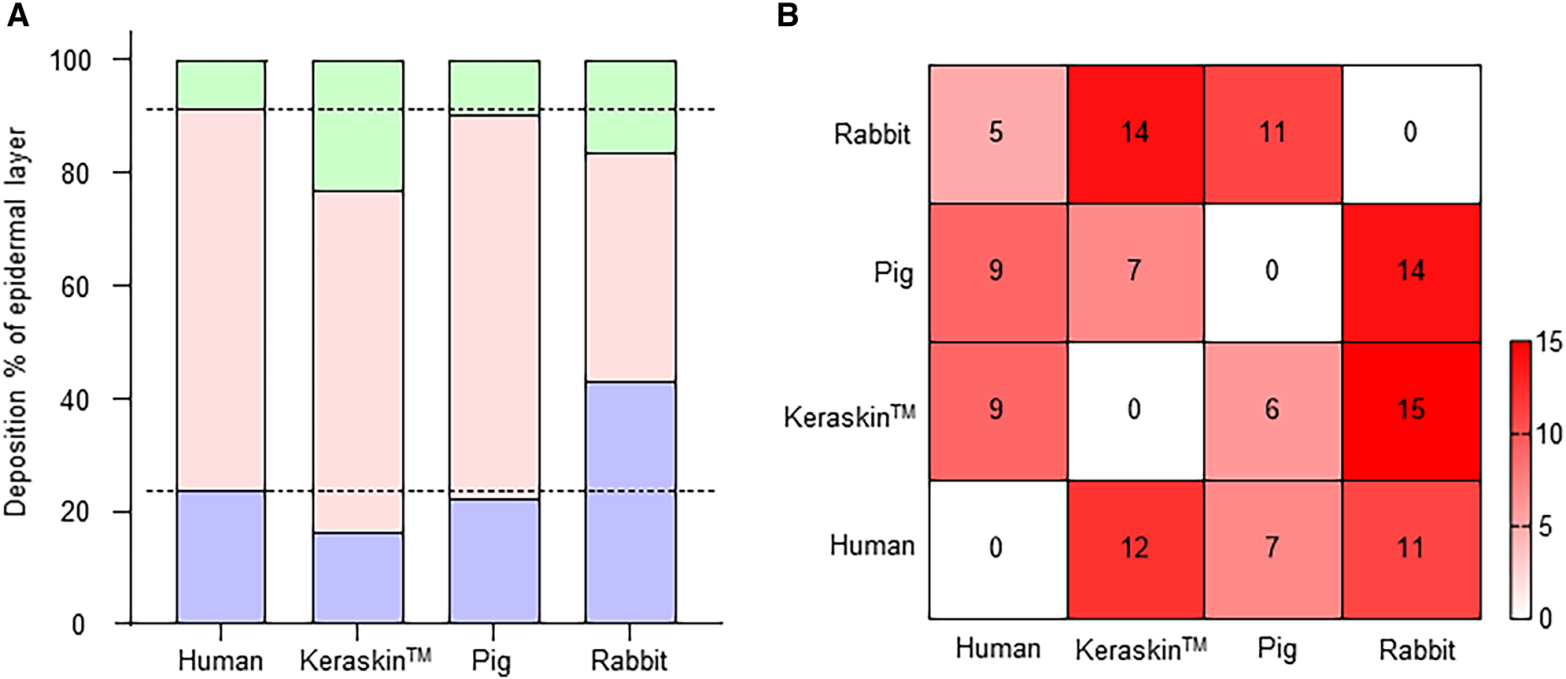
Measurement of similarity between each model. **A** Deposition percentage of epidermal layers of each model. The percentage was calculated by comparing the sum of the average thickness of the granular, spinous, and basal layers with the average thickness of each layer. **B** Heatmap corresponding to the scoring data. The definition of similarity scoring is provided in Table 1. The left heatmap belongs to each model, and the right heatmap belongs to the other models. A deeper red color indicates lower similarity to the compared model

Considering that epidermal thickness differed among the skin surrogates, the relative thickness of the epidermal layers was compared anatomically. The epidermal structure of Keraskin showed a relatively higher proportion of the granular layer than the other models (Fig. 3A). Similarly, the rabbit skin also showed a higher proportion of the granular layer than found in the human or pig skin. Among the examined skin surrogates, the pig skin showed the highest structural similarity to the human skin, with minimal differences (Table 1 and Fig. 3A). Next, we ranked and scored the similarity between skin models based on thickness scored on a scale of 3 (1, closest; 2, moderate; 3, furthest). The pig skin was the most similar to the human skin, with a total of 7 points (closest 5, furthest 15), whereas Keraskin was the most different, with a total score of 12 points (Fig. 3B). Interestingly, the pig skin and Keraskin were more similar to each other than to human skin, with scores of 6 and 7, respectively (Table 1 and Fig. 3B). The rabbit skin was the least similar to the human skin among the three models (Table 1 and Fig. 3B). Taken together, pig skin was identified as the surrogate model with the most similar epidermal structure to that of human skin.

**Table 1:** Assessment of similarity scores between skin models

Comparison^†^	Human (H)			Keraskin (K)			Pig (P)			Rabbit (R)		
	K	P	R	H	P	R	H	K	R	H	K	P
Epidermis	3	1	2	2	1	3	2	1	3	1	3	2
Cornified layer	3	1	2	2	1	3	1	3	2	1	3	2
Granular layer	3	2	1	2	1	3	2	1	3	1	3	2
Spinous layer	2	1	3	2	1	3	2	1	3	1	3	2
Basal layer	1	2	3	1	2	3	2	1	3	1	2	3
Total	12	7	11	9	6	15	9	7	14	5	14	11

^†^A comparison was performed for each skin model (top row) in relation to each other (second row). The similarity between the skin models based on thickness was scored on a scale of 3. The scale of 3 was ranked in ascending order as score by comparing the absolute values. The absolute value was calculated by subtracting the average thickness of comparison groups to that of each skin model. The lowest absolute value was scored as 1, the second absolute value as 2, and the highest absolute value as 3. A lower score indicates greater similarity than the other models

### Comparison of epidermal barrier marker expression in surrogate skin models and human skin

We further examined the expression of human FLG, CLDN1, and CDH1 in the candidate skin surrogates to compare the strength of the epidermal barrier. Keraskin, which is produced using primary human epidermal keratinocytes, expressed all three markers, similar to human skin. In contrast, the pig skin was negative for FLG staining and the rabbit skin was negative for all three human epidermal markers (Fig. S1). Although this result may stem from species differences, it suggests that Keraskin may be the most similar to human skin at the molecular level.

### Proliferation index of human skin surrogate models

**Fig. 4:**
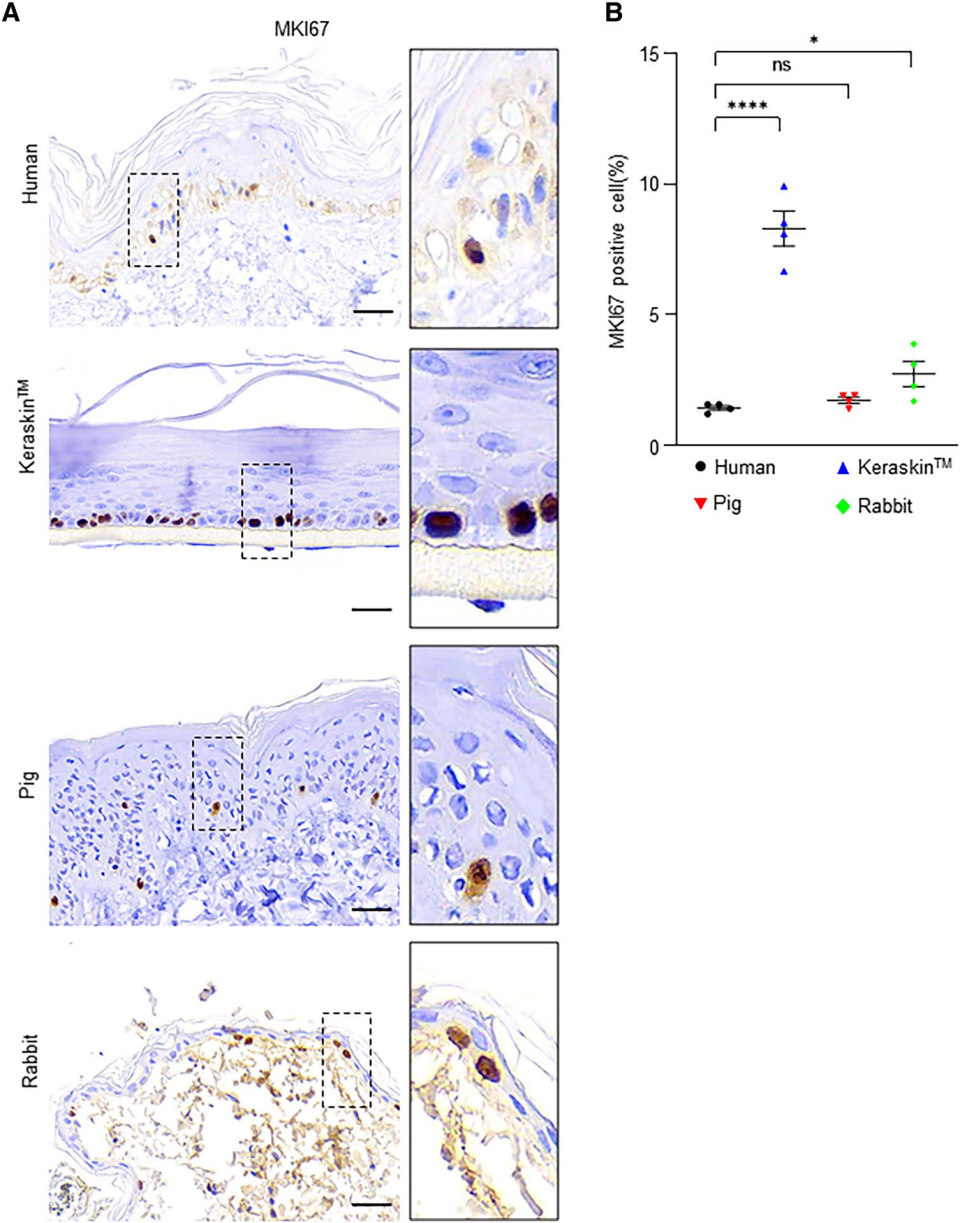
Keratinocyte proliferation in each model. **A** Immunohistochemistry staining images of human skin, Keraskin, pig skin, and rabbit skin for the proliferation index MKI67. Scale bars = 30 µm. **B** Quantification of the percentage of keratinocytes expressing MKI67 antigen. Data are presented as the mean ± SEM. ns, not significant (*p* > 0.05); *p < 0.05, ****p < 0.0001; unpaired Student’s t test, n = 20

The proliferation index of basal keratinocytes in each skin model was evaluated using nuclear staining for MKI67, a universally accepted proliferation marker (Fig. 4A). The proliferation index of the pig skin (1.73%) was not significantly different from that of human skin (1.45%), whereas that of the rabbit skin was slightly higher (2.75%) than that of human skin (Fig. 4B). Interestingly, the proliferation index was estimated to be 8.47% for Keraskin, which was significantly higher than that of human skin (Fig. 4B).

## Discussion

The epidermis is divided into four layers: the cornified, granular, spinous, and basal layers [16]. Each layer of the epidermis plays an important role in the formation of the epidermal barrier and the physiology of the skin. In particular, the stratum corneum is the key to maintaining skin barrier function and xenobiotic metabolism [17]. Accordingly, the structural integrity of the stratum corneum is closely linked to resistance to skin irritation [18] and the development of skin diseases [19]. The epidermis of RHE [14] and ex vivo skin models from animals consists of the same epidermal layers as found in humans [20]. However, few studies have compared the epidermal structures of each skin model with those of human skin in detail.

We compared the thickness of each epidermal layer to identify which of the three skin models has an epidermal structure that is most similar to that of human skin. The results demonstrated that pig skin is structurally the most similar to human skin. In contrast, the rabbit skin was the least similar to human skin, displaying the thinnest stratum corneum, granular layer, and epidermis. Rabbit skin is characterized by a thin epidermis [9] composed of only one or two cell layers. We confirmed that the deposition percentage of the epidermis was significantly lower than that of human skin. In contrast, the proliferation index of the rabbit skin was much higher than that of the human skin.

It is well known that rabbits exhibit higher sensitivity to skin irritants than humans [3]. Of the 16 chemicals identified as irritants using skin tests in rabbits, only five showed irritation in the human patch test, resulting in 56% concordance. In contrast, the RHE models EpiDerm and Episkin showed concordance of 76% and 70% with the human patch test, respectively. RHE models were developed to resemble the human stratum corneum and epidermis with respect to thickness and lipid composition [21], with the goal of enabling their use in SITs to identify human skin irritants. Indeed, we found that Keraskin, an RHE model approved in OECD TG 439 for *in vitro* skin irritation tests, showed a thicker stratum corneum and epidermis than those of the human and animal skin samples. We speculate that a thicker stratum corneum is needed to compensate for the high skin permeability, which could ultimately improve the utility of the RHE model in predicting irritants to human skin.

Indeed, skin permeability and barrier function are important factors in the SIT [22]. In addition, Keraskin showed a high proliferation index; therefore, the recovery pattern from irritation is expected to be higher with this RHE model than for human skin. This may stem from the short period of culturing Keraskin in growth factor-rich media. RHE models show higher trans-epidermal water loss than human skin, which suggests higher skin permeability [23]. Furthermore, Keraskin has no dermis or rete ridges and pars papillaris, unlike human and pig skin [12], suggesting that there are certain limitations of the RHE in mimicking the epidermal structure of the human skin.

We demonstrated that ex vivo pig skin most closely resembles human skin in physiological, histological, and structural aspects, which logically supports its use as a surrogate model for human skin. Ex vivo pig skin can be ethically obtained as a by-product from the slaughterhouse. However, not all human skin barrier proteins are expressed in pig skin, which is a drawback compared to the use of RHE models. As a further experiment, comparing the results of SITs between pig skin and RHE models could offer a good opportunity to broaden the interpretation of the results for inferring skin hazards of chemicals to humans in cosmetics development.

## Supplementary Information

Below is the link to the electronic supplementary material.

Supplementary file1 (TIF 711 kb) Supplementary Figure 1. Immunohistochemistry (IHC) staining images for each model. (A) IHC staining images of human skin, Keraskin, pig skin, and rabbit skin for FLG, CLDN1, and CDH1. Scale bars = 30 µm. This figure shows the epitope availability of the antibody using the human antigen for IHC. Keraskin can be stained by all antibodies. FLG is not stained in the pig skin. FLG, CLDN1, and CDH1 are not stained in the rabbit skin

## Data Availability

Data sharing will be considered by the corresponding author (Ki Taek Nam) upon request.
