# Supplementary figures and images for: Comparison of structural characteristics and molecular markers of rabbit skin, pig skin, and reconstructed human epidermis for an ex vivo human skin model
Source: Toxicol Res. 2023 May 4;39(3):477–84. doi: 10.1007/s43188-023-00185-1 (PMC10313609; doi:10.1007/s43188-023-00185-1)

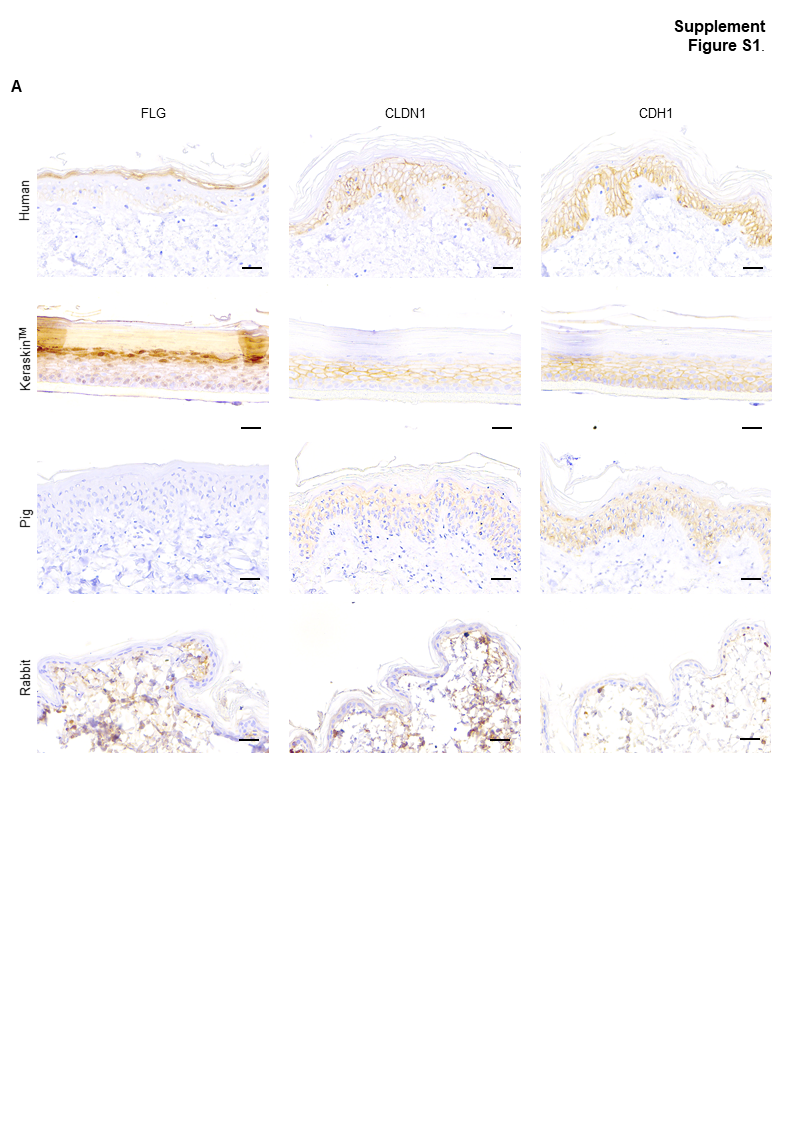

Supplement: Supplementary file 1 — Supplementary file1 (TIF 711 kb) Supplementary Figure 1. Immunohistochemistry (IHC) staining images for each model. (A) IHC staining images of human skin, Keraskin, pig skin, and rabbit skin for FLG, CLDN1, and CDH1. Scale bars = 30 µm. This figure shows the epitope availability of the antibody using the human antigen for IHC. Keraskin can be stained by all antibodies. FLG is not stained in the pig skin. FLG, CLDN1, and CDH1 are not stained in the rabbit skin [file 43188_2023_185_MOESM1_ESM.tif]
